# Valorization of Liquor Waste Derived Spent Coffee Grains for the Development of Injection-Molded Polylactide Pieces of Interest as Disposable Food Packaging and Serving Materials

**DOI:** 10.3390/foods11081162

**Published:** 2022-04-16

**Authors:** Enrique Terroba-Delicado, Stefano Fiori, Jaume Gomez-Caturla, Nestor Montanes, Lourdes Sanchez-Nacher, Sergio Torres-Giner

**Affiliations:** 1 Technological Institute of Materials (ITM), Universitat Politècnica de València (UPV), Plaza Ferrándiz y Carbonell 1, 03801 Alcoy, Spain; enterde@epsa.upv.es (E.T.-D.); jaugoca@epsa.upv.es (J.G.-C.); nesmonmu@upvnet.upv.es (N.M.); lsanchez@mcm.upv.es (L.S.-N.); 2 R&D Department, Condensia Química S.A., Calle de la Cierva 8, 08184 Palau-soilità i Plegamans, Spain; sfiori@condensia.com; 3 Research Institute of Food Engineering for Development (IIAD), Universitat Politècnica de València (UPV), Camino de Vera s/n, 46022 Valencia, Spain

**Keywords:** PLA, coffee liquor, food packaging materials, waste valorization, Circular Bioeconomy

## Abstract

The present work puts the Circular Bioeconomy’s concept into action, originally valorizing residues of spent coffee grains from the beverage liquor coffee industry to develop green composite pieces of polylactide (PLA). The as-received spent coffee grains were first milled to obtain the so-called spent coffee grounds (SCGs) that were, thereafter, incorporated at 20 wt.% into PLA by extrusion. Finally, the resultant green composite pellets were shaped into pieces by injection molding. Moreover, two oligomers of lactic acid (OLAs), namely OLA2 and OLA2_mal_, the latter being functionalized with maleic anhydride (MAH), were added with SCGs during the extrusion process at 10 wt.%. The results show that, opposite to most claims published in the literature of green composites of PLA, the incorporation of the liquor waste derived SCGs increased the ductility of the pieces by approximately 280% mainly due to their high lipid content. Moreover, the simultaneous addition of OLA2 and OLA2_mal_ further contributed to improve the tensile strength of the green composite pieces by nearly 36% and 60%, respectively. The higher performance of OLA2_mal_ was ascribed to the chemical interaction achieved between the biopolyester and the lignocellulosic fillers by the MAH groups. The resultant green composite pieces are very promising as disposable food-serving utensils and tableware.

## 1. Introduction

In recent years, society has become more aware about the environmental issues produced by the extensive use of petrochemical polymers that are not compostable or recyclable. This has promoted the development of environmentally friendly polymer materials that can potentially replace conventional polymers [1]. Among these materials, green composites are particularly interesting, since they are formed by a biopolymer matrix and a natural filler [2,3,4]. Furthermore, green composite materials can strongly contribute to the development of the so-called “Circular Bioeconomy”, since these are obtained from natural resources or, more sustainably, wastes and, after use, can fully disintegrate under different composting conditions [5]. In the area of biopolymers, polylactide (PLA) has made a large contribution, since this biopolyester is both fully bio-based, obtained from starch-rich materials, and compostable in controlled industrial facilities [6,7]. PLA is a linear biopolyester that shows high mechanical resistance and thermal stability, good transparency, and water-barrier performance. This thermoplastic shows a high application interest in food packaging [8,9,10]; however, in spite of this, the use of PLA-based articles in large-scale applications is still limited by its poor toughness and brittle behavior, low thermomechanical resistance, and relatively high cost [11]. In particular, its inherent high brittleness entails a considerable scientific challenge in the food packaging field [12].

In the novel Circular Bioeconomy context, lignocellulosic materials obtained from processing by-products and wastes of the food and agroforestry industries are also gaining more attention as cost-effective fillers due to their natural origin and favored biodegradability [13]. Thus, several agri-food waste derived fillers have been reported in recent years, for example, almond shell [14], peanut skin [15,16], argan nut shell [17,18], pineapple flour [19], coconut fibers [20], banana and plantain fibers [21], or pinecone [22]. In this regard, large quantities of by-products and wastes are generated during the ‘fruit-to-cup’ transformation of coffee beans, not only including leaf, flower, and cherry, but also coffee husks, coffee silver skin (CSS), and spent coffee grounds (SCGs) [23,24]. SCGs are the resultant dark-colored residual materials obtained during the treatment of coffee powder with hot water or steam for the instant (or hydrosoluble) coffee preparation. In this regard, it is considered that nearly 50% of the coffee production worldwide is processed for soluble coffee preparation, whereas only 0.33–0.45 kg of instant coffee is obtained from 1 kg of green coffee beans in the soluble coffee industry [25]. As a result, this process generates 550–670 g of SCGs per kg of coffee beans, which is equivalent to 6 million tons of SCGs per year. Polysaccharides are the most abundant components in SCGs (12.4 wt.% cellulose, 39.1 wt.% hemicellulose, and 23.8 wt.% lignin), followed by proteins (17.4 wt.%), lipids (2.3 wt.%), and ashes (1.3 wt.%, mainly potassium minerals), which varies depending on both the variety of coffee beans and extraction process [26]. However, the coffee liquor industry is not so popular due to the fact that it is limited to specific geographical areas. Liquor, also known as spirit, is an alcoholic beverage produced by the distillation of plants or grains, which have already experienced alcoholic fermentation. In this industry, coffee grains are naturally roasted and then macerated with ethanol to obtain the alcoholic beverage. Furthermore, unlike instant coffee, which uses pressurized hot water (~175 °C) to extract all of the soluble solids and volatile compounds [23], the coffee used in the liquor industry is not subjected to any drastic temperature or extraction processes. Therefore, the resultant SCGs can retain their natural compositions in proteins and lipids. The potential uses of SCGs include biodiesel production [27], elements to increase compost quality [28], water purification of pharmaceutic products [29], or as a biofuel on its own [30]. Nevertheless, SCGs can also be used as reinforcing fillers in the development of green composites [31,32,33]. Moreover, SCGs also contain polyphenols and free radicals that can offer antioxidant and anti-tumor activities [34]. However, similar to other lignocellulosic fillers or fibers, these tend to act as reinforcing elements due to their relatively high hardness. Thus, when they are incorporated into biopolymer matrices, hardness is increased, but the ductile properties are noticeably reduced [35].

Furthermore, one of the main drawbacks of lignocellulosic fillers when incorporated into polymer matrices is their poor filler-matrix interfacial adhesion, which increases the probability of particle aggregate formation. This low compatibility is based on the their poor chemical affinity, since PLA and other biopolyesters are highly hydrophobic, while lignocellulosic fillers present a more hydrophilic character [36]. As a result of this poor affinity, the thermal stability and mechanical strength and ductility of the resultant green composites of PLAs tend to decrease. In this regard, the use of plasticizers or compatibilizers can be very useful for improving the interfacial adhesion between the lignocellulosic fillers and polymer matrices [37]. In green composites, compatibilization can be achieved through a melt-grafting process of the natural fillers onto the polymer chains during extrusion using multi-functional additives, that is, with multiple reactive groups or sites [38]. Normally, these reactive compatibilizers are based on low-molecular-weight (M_W_) polymers or oligomers containing multiple epoxy or maleic anhydride (MAH) groups [39]. During the so-called reactive extrusion (REX), some of the oxygen-based groups of the compatibilizer form new ester bonds with the terminal hydroxyl (—OH) or carbonyl (—COOH) groups of the biopolyester while, at the same time, other groups can react with the —OH groups present on the surface of the lignocellulosic filler, improving the compatibility by the formation of covalent bonds [14,40]. In PLA formulations, the oligomers of lactic acid (OLAs) can habitually favor compatibility through the plasticization of the biopolyester matrix, taking advantage of their similar chemical structure. In particular, 20 wt.% OLAs have been reported to reduce the glass transition temperature (T_g_) of PLAs by 13 °C [41] as well as improve its mechanical ductile properties [42]. Moreover, it is possible to chemically modify OLAs by incorporating reactive functional groups into their structure. For example, Lowe et al. [43] reported the development of controlled M_W_ telechelic lactic acid oligomers with acrylate functionalities by transesterification. Additionally, the work of Grosse et al. [44] reported the addition of functional MAH groups to the structure of OLAs. Therefore, the resultant chemically modified OLAs are expected to serve as potential reactive compatibilizers for blends or composites based on PLA.

This work reports the preparation and characterization of injection-molded pieces of PLA/SCG green composites with the objective of developing a sustainable and cost-efficient material for food packaging and food contact disposables. To improve the compatibility between the PLA matrix and the SCG filler, and then the performance of the green composite, two novel OLAs were originally tested, namely OLA2 and OLA2_mal_. Whereas OLA2 is designed to improve the impact strength of PLA-based formulations, OLA2_mal_ is a chemically modified grade that presents several reactive MAH groups distributed along the oligomer chains.

## 2. Materials and Methods

### 2.1. Materials

PLA, with the commercial reference PURAPOL L130 grade and based on a 99% _L_-isomer, was provided by Corbion (Gorinchem, The Netherlands). It shows a density of 1.24 g·cm^−3^ and a melt flow rate (MFR) of 16 g/10 min (210 °C and 2.16 kg). This PLA grade is granted with the food contact status, whereas it is suitable for injection molding and is in compliance with the EN 13432 standard to be processed by industrial composting.

**Figure 1:**
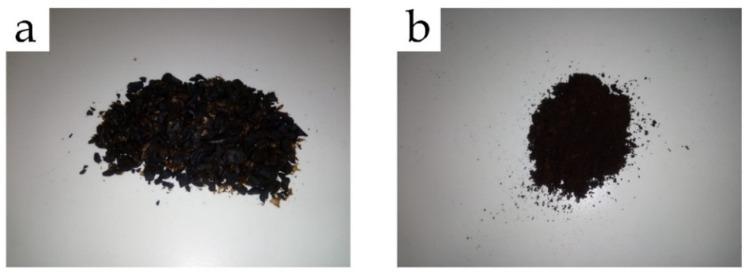
(**a**) As-received spent coffee grains from the production of liquor coffee, and (**b**) the resultant spent coffee grounds (SCGs) obtained after washing, drying, and milling.

The spent coffee grains were supplied by Licores Sinc. S.A. (Alcoy, Spain), which is obtained as a by-product from the production of liquor coffee. In this process, coffee grains were first naturally roasted and then macerated for 10 days in alcohol to obtain the drink called coffee liquor. The received biomass obtained after the process of maceration was washed several times with clean water and, subsequently, vacuum dried in a dehumidifying stove (MCP Vacuum Casting System, Lubeck, Germany) for 1 week at 60 °C. Finally, to obtain the so-called SCGs, the grains were milled in a ZM 200 centrifugal mill from Retsch (Düsseldorf, Germany) at a speed of 12,000 rpm and sieved with a 250 µm mesh filter. Figure 1 presents the images of the as-received liquor waste-derived spent coffee grains (Figure 1a) and the resultant SCG particles (Figure 1b).

**Figure 2:**
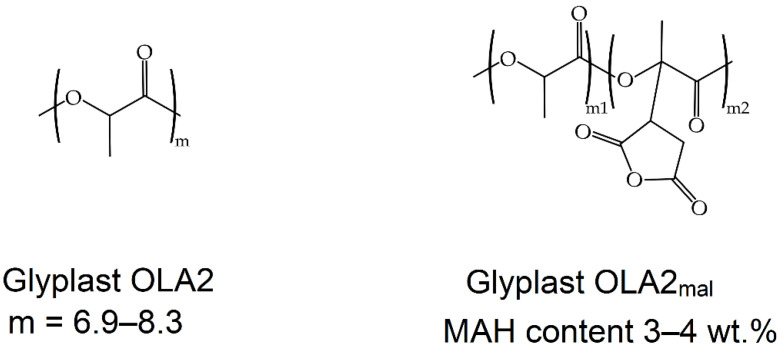
Chemical structures of the oligomers of lactic acid (OLAs): Gyplast OLA2 (**left**) and Gyplast OLA2_mal_ (**right**).

Glyplast OLA2 and OLA2_mal_ were provided by Condensia Química Inc. (Barcelona, Spain). Glyplast OLA2 is a liquid oligomer of lactic acid with an M_W_ comprised between 500–600 g·mol^−1^. It has a viscosity of 90 mPa s at 40 °C and an ester content of >99%. Its density is 1.10 g·cm^−3^, it has a maximum acid number of 2.5 mg KOH·g^−1^, and a maximum water content of 0.1 wt.%. With regard to Glyplast OLA2_mal_, it is an OLA2 derivative grafted with MAH. The MAH content ranges between 3 and 4 wt.%. Figure 2 depicts the chemical structure of both oligomers.

### 2.2. Preparation of the PLA/SCG Composites

The PLA pellets and both OLAs, that is, OLA2 and OLA2_mal_, were initially dried at 60 °C and 40 °C, respectively, for 48 h in a dehumidifying dryer MDEO to remove any residual moisture prior to processing. Then, the corresponding weight fraction (wt.%) of each component was mixed and pre-homogenized in a zipper bag. The corresponding formulations, shown in Table 1, were compounded in a twin-screw extruder from Construcciones Mecánicas Dupra, S.L. (Alicante, Spain). This extruder has a 25 mm diameter with a length-to-diameter ratio (L/D) of 24. The extrusion process was carried out at a rate of 22 rpm, using the following temperature profile (from hopper to die): 180–185–190–195 °C. The compounded materials were pelletized using an air-knife unit. In all cases, the residence time was approximately 1 min.

The resultant pellets after the extrusion process were shaped into standard samples in a Meteor 270/75 injector from Mateu & Solé (Barcelona, Spain). The temperature profiles in the injection molding unit were 185 °C (hopper), 190 °C, 195 °C, and 200 °C (injection nozzle). A clamping force of 75 tons was applied, while the cavity filling and cooling times were set to 1 and 10 s, respectively. Standard samples, with an average thickness of 4 mm, were obtained for characterization.

### 2.3. Characterization of the PLA/SCG Composite Pieces

#### 2.3.1. Morphological Characterization

The morphology of the SCG particles and fractured samples obtained from the Charpy tests were studied by field emission scanning electron microscopy (FESEM) in a ZEISS ULTRA 55 microscope from Oxford Instruments (Abingdon, UK). Prior to observation, the samples were sputtered with a gold-palladium alloy in an EMITECH sputter coating SC7620 model from Quorum Technologies, Ltd. (Laughton, UK). The FESEM measurements were carried out using an acceleration voltage of 2 kV.

#### 2.3.2. Mechanical Characterization

The tensile properties of PLA/SCG pieces sizing 150 mm × 10 mm × 4 mm were obtained using a universal testing machine ELIB 50 from S.A.E. Ibertest (Madrid, Spain), as recommended by ISO 527-1:2012. A 5-kN load cell was used and the cross-head speed was set to 5 mm·min^−1^. The shore hardness was measured in a 676-D durometer from J. Bot Instruments (Barcelona, Spain), using the D-scale on rectangular samples with the dimensions of 80 mm × 10 mm × 4 mm and according to ISO 868:2003. The impact strength was also studied by means of injection-molded rectangular samples with the dimensions of 80 mm × 10 mm × 4 mm in a Charpy pendulum (1-J) from Metrotec S.A. (San Sebastián, Spain) on notched samples (0.25 mm radius V-notch), following the specifications of ISO 179-1:2010. All of the mechanical tests were performed at room temperature, and 6 samples of each material were tested and the corresponding values were averaged.

#### 2.3.3. Thermal Analysis

The most relevant thermal transitions of the PLA/SCG green composites were obtained by differential scanning calorimetry (DSC) in a Mettler-Toledo 821 calorimeter (Schwerzenbach, Switzerland). The samples with an average weight of 6–7 mg were subjected to a thermal program divided into 3 stages: an initial heating from 25 °C to 180 °C, followed by a cooling to 0 °C, and a second heating to 300 °C. All the heating and cooling rates were set at 10 °C·min^−1^ and the tests were run in a nitrogen atmosphere with a flow-rate of 66 mL·min^−1^ using standard sealed aluminum crucibles with a capacity of 40 μL. The percentages of the crystallinity (X_c_) and maximum crystallinity (X_c_max_) were calculated during the second heating using the following equations:

(1) $$X_{c}=\left[\frac{∆H_{m}-∆H_{\mathrm{cc}}}{∆H_{m}^{0}\times \left(1-w\right)}\right]\times 100$$

(2) $$X_{c\_\max}=\left[\frac{∆H_{m}}{∆H_{m}^{0}\times \left(1-w\right)}\right]\times 100$$

where $∆H_{m}^{0}=93.7$ J/g is the theoretical enthalpy of a 100% crystalline PLA sample [45], the term 1 − w corresponds to the PLA weight fraction in the blend, and $∆H_{m}$ and $∆H_{\mathrm{cc}}$ are, respectively, the melting and cold crystallization enthalpies.

The thermal degradation of the PLA/SCG pieces was assessed by thermogravimetric analysis (TGA) in a LINSEIS TGA 1000 (Selb, Germany). The samples with a weight ranging from 15 and 17 mg were placed in 70 µL alumina crucibles and subjected to a dynamic heating program from 40 °C to 700 °C at a heating rate of 10 °C·min^−1^ in an air atmosphere. The first derivative thermogravimetric (DTG) curves were also determined. All of the thermal tests were carried out in triplicate in order to obtain reliable results.

#### 2.3.4. Thermomechanical Characterization

Dynamical mechanical thermal analysis (DMTA) was carried out in a DMA1 dynamic analyzer from Mettler-Toledo, working in single cantilever flexural conditions. Rectangular samples with the dimensions of 20 mm × 6 mm × 2.7 mm were subjected to a dynamic temperature sweep from 30 °C to 140 °C at a constant heating rate of 2 °C·min^−1^. The selected frequency was 1 Hz and the maximum flexural deformation or cantilever deflection was set to 10 µm. All of the thermomechanical tests were performed in triplicate.

#### 2.3.5. Color and Wetting Characterization

A Konica CM-3600d Colorflex-DIFF2 from the Hunter Associates Laboratory, Inc. (Reston, VA, USA) was used for the color measurement. The color coordinates (L*a*b*) were measured, with the illuminant D65 and observer 10°, according to the following criteria: L* = 0, darkness; L* = 100, lightness; a* represents the green (a* < 0)-to-red coordinate (a* > 0); and b* represents the blue (b* < 0)-to-yellow coordinate (b* > 0). The tests were run in triplicate.

Contact angle measurements were performed with an EasyDrop Standard goniometer model FM140 (KRÜSS GmbH, Hamburg, Germany), which was equipped with a video capture kit and analysis software (Drop Shape Analysis SW21; DSA1). Double distilled water was used as the test liquid. The tests were replicated 10 times.

#### 2.3.6. Water Absorption Test

The water absorption capacity of the PLA/SCG pieces was evaluated by the water uptake method [37]. Injection-molded samples sizing 80 mm × 10 mm × 4 mm were first weighted in a balance and then placed inside a beaker filled with distilled water. The samples were wrapped with a metal grid. Subsequently, the samples were removed from the beaker and weighed at different time intervals for up to 14 weeks. For every measurement, the superficial moisture of the samples was removed using tissue paper. Three samples of each formulation were tested.

#### 2.3.7. Biodisintegration Test

The biodegradability of the samples was evaluated using a disintegration test in controlled compost conditions, according to the guidelines of the ISO 20200 standard at the conditions of 58 °C and a relative humidity (RH) of 55%. Injection-molded samples with the dimensions of 30 mm × 30 mm × 4 mm were placed in a carrier bag and buried in soil with the following controlled composition: sawdust (40 wt.%), rabbit-feed (30 wt.%), ripe compost (10 wt.%), corn starch (10 wt.%), saccharose (5 wt.%), corn seed oil (4 wt.%), and urea (1 wt.%). To monitor the biodisintegration process, the samples were periodically unburied, washed with distilled water, dried, and weighed in an analytical balance. Pictures of the samples were also taken at each weight measurement, with the objective to visually evaluate the biodisintegration process. The weight loss, as a result of the disintegration in the controlled compost soil, was calculated by means of the following expression:

(3) $$\mathrm{Weight}\;\mathrm{loss}\;\left(\%\right)=\left(\frac{W_{0}-W_{t}}{W_{0}}\right)\times 100$$

where W_t_ is the weight of the sample after a certain period of time in the controlled compost soil and W_0_ is the initial dry weight of the sample. All of the tests were triplicated in order to ensure the reliability of the results.

### 2.4. Statistical Analysis

The results were subjected to analysis of variance (ANOVA) using Statgraphics Centurion XVII-64 software (Manugistics Corp., Rockville, MD, USA). To this end, significant differences were assumed with a significance level greater than 95% (*p* < 0.05).

## 3. Results and Discussion

### 3.1. Morphology of the SCG Particles

**Figure 3:**
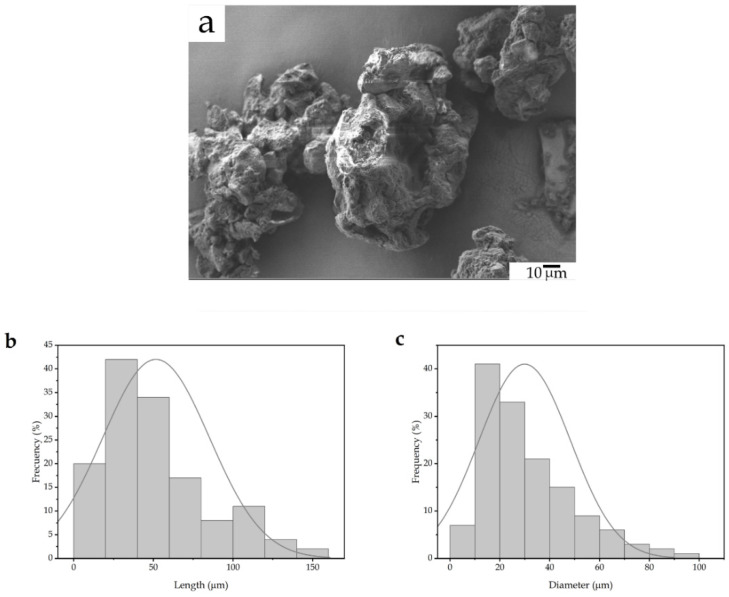
(**a**) Field emission scanning electron microscope (FESEM) image of the spent coffee ground (SCG) particles. Image taken with a magnification of 500× and a scale marker of 10 µm; (**b**) Histogram of the lengths of the SCG particles; and (**c**) histogram of the diameters of the SCG particles.

Particle interlocking is an essential mechanism in polymer composites. Figure 3 shows the morphology of the SCG particles observed by FESEM and their respective length and diameter histograms. In Figure 3a, one can observe that the lignocellulosic particles display a round-like morphology with a rough surface, which can be ascribed to the effect of the milling process on the particles with high hardness. These particles also displayed high porosity on their surface, which can be beneficial towards interactions with the biopolymer matrix by acting as anchoring points. In this regard, Mendes et al. [46] observed a very similar structure for the SCG powder when studying the composites of high-density polyethylene (HDPE). It was also reported that SCG particles tend to form small aggregates due to their hydrophilic characteristic. In this regard, Garcia-Garcia et al. [33] expressed that the individual particle size is in the 15–20 μm range, whereas these form aggregates sized 60–80 μm. Figure 3b,c present the collation of the particle histograms in terms of the length and diameter, respectively. The particles presented an average length of approximately 42.5 µm, whereas the average diameter was ~40 µm. In both cases, the dimensions were defined by a classical monomodal distribution. Furthermore, this particle morphology is based on a relatively low size, which can offer a positive effect on the overall properties. For instance, Crespo et al. [47] observed that large lignocellulosic particles, with diameter sizes exceeding 150 µm, led to an impairment and certain heterogeneity in the composites of vinyl plastisols.

### 3.2. Optical Properies of the PLA/SCG Composites

**Figure 4:**
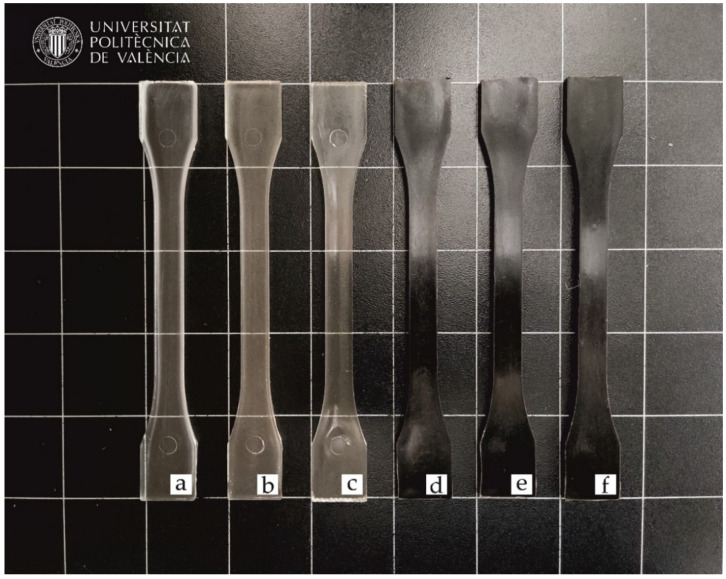
The visual appearance of the injection-molded pieces of the polylactide (PLA)/spent coffee grounds (SCGs) containing the oligomers of lactic acid (OLAs): (**a**) PLA; (**b**) PLA + OLA2; (**c**) PLA + OLA2_mal_; (**d**) PLA + SCG; (**e**) PLA + OLA2 + SCG; and (**f**) PLA + OLA2_mal_ + SCG.

The visual appearance is essential in terms of the impression that an end-product makes on the consumer. In this sense, Figure 4 shows the visual appearance of the pieces of PLA and its combinations with SCGs and the two-tested OLAs obtained by injection molding. It can be observed that the neat PLA piece and the pieces of PLA processed with both OLAs showed a relatively high contact transparency. The loss of transparency in the PLA sample is due to the semicrystalline nature of the biopolyester, in which the amorphous and crystalline regions have different light refraction indexes [48]. While the addition of OLA2 and OLA2_mal_ kept the original appearance of the PLA piece, the samples containing SCGs developed a dark brown-to-black tonality. The latter optical effect is due to the intrinsic characteristic color of these SCG particles, as it can be observed in Figure 1, making the samples also completely opaque.

Table 2 gathers the L*a*b* color coordinates obtained for these injection-molded pieces. Luminance (L*) is indicative of the brightness or lightness of the color of a sample. In this sense, the injection-molded PLA piece and the PLA/OLA2 and PLA/OLA2_mal_ pieces exhibited very similar L* values, in the range of 45–48. This was expected due to the very similar nature and miscibility between the biopolymer and its oligomers. In the case of the green composite pieces, which were filled with SCGs, these presented significantly lower (*p* < 0.05) L* values than PLA, of approximately 26, not being significantly different (*p* > 0.05) among the two samples. Furthermore, the color coordinate a*, which is representative of a green (negative) or red color (positive), showed values in the range of −0.23 to 0.13 for the pieces of PLA, PLA/OLA2, and PLA/OLA2_mal_. In the case of b*, which indicates a blue (negative) or yellow (positive) color, all of the unfilled pieces presented similar positive values between 1 and 2. Therefore, these pieces tend to show a slight yellow, pale color, showing no significant differences (*p* > 0.05) among them. On the contrary, all the SCG-containing pieces showed very positive a* values, in the range of 0.7–1.1, whereas the b* values were in the 4.9–5.6 range. The combination of the red and yellow colors thus quantified the above-qualified dark brown aspect of the green composite pieces. A similar color change was reported by Suaduang et al. [31] for the PLA/SCG composites containing up to 10 wt.% SCGs, showing a* and b* values from −0.9 to 7.5 and from −4.4 to 16.5, respectively.

### 3.3. Mechanical Properties of the PLA/SCG Composites

Table 3 gathers the results obtained in the mechanical characterization of the PLA/SCG pieces. These results are of great interest to evaluate the effect of both SCGs and OLA2 and OLA2_mal_ on PLA in terms of the mechanical resistance and ductile properties, which can be relevant for food packaging applications. It can be observed that the neat PLA piece showed a Young modulus (E) of 2913 MPa, a maximum tensile strength (σ_max_) of 52.4 MPa, and an elongation at break (ε_b_) of 10.4 %. These values agree with those previously reported by, for instance, Agüero et al. [49]. These are indicative of a rigid material with low ductility, particularly when compared to other biopolymer pieces, such as those of bio-based high-density polyethylene (bio-HDPE) [50] or polyethylene terephthalate (bio-PET) [51], showing values of ε_b_ of nearly 500%. The incorporation of the SCG particles into PLA decreased all of the mechanical resistance properties. Then, the E and σ_max_ values were reduced to 2367 MPa and 13.9 MPa, respectively, which indicates that a poor dispersion of the lignocellulosic fillers in the biopolyester matrix was attained [32]. However, contrary to most of the claims published in the literature of PLA composites based on lignocellulosic fillers derived from soluble coffee wastes [52], the ductile properties were improved. In particular, the ε_b_ value significantly increased to 39.6%, which approximately corresponds to a 280% increase in relation to the neat PLA piece. This ductility improvement suggests that the PLA matrix was plasticized by the SCG particles, since this waste-derived biomass can contain traces of water and ethanol and, more importantly, large amounts of lipids [53]. Among the lipids, the main plasticizing molecules correspond to organic compounds containing oxygen-based groups, such as fatty acids [54]. In any case, since both water and ethanol were removed by drying prior to processing the samples by extrusion to avoid the hydrolysis of PLA, plasticization can then be mainly ascribed to the presence of fatty acids. In this regard, it has been reported that the coffee oil content in SCGs is 10.98 wt.%, from which approximately 46% corresponds to linoleic acid [55]. In this sense, the work of Battegazzore et al. [56] reported the presence of several plasticizing compounds in natural fillers, such as hazelnut skin or cocoa by-products, which were also used to successfully increase the ductility of PLA.

From the above, it can be considered that coffee wastes derived from the liquor industry seem to possess a higher lipid content than those obtained from traditional soluble coffee. This key effect is related to differences in their processing technologies that, in the case of coffee liquor, does not imply the use of high temperature or extraction steps, apart from the natural roasting of the coffee grains. This result agrees with the findings reported by Suaduang et al. [31], who showed that contents of 7.5 and 10 wt.% of SCGs derived from the coffee bean roasting process, not yet being used for soluble or liquor coffee, resulted in a slight increase in the elongation at break of PLA films. In this former study, ε_b_ increased from 4.18% and 5.04% in the transversal (TD) and machine direction (MD), to 4.24% and 5.31% and to 5.33% and 6.63% for 7.5 and 10 wt.% loadings of SCGs, respectively. The higher improvement observed here can be then related to the higher SCG content used in the green composite, but also, more importantly, to the potential differences in the high fatty-acid content and remaining solvent traces due to the particular production process of coffee liquor and mild drying conditions. Indeed, as described above, other more conventional polymer composites based on SCGs derived from the soluble coffee industry showed that increasing the filler content reduced the ductility of the polymer. For instance, the work of Mendes et al. [46] reported a decrease in ε_b_ of HDPE of approximately 50%, in relation to neat HDPE, for a 30 wt.% SCG content.

In relation to the two tested OLAs, the addition of OLA2 and OLA2_mal_ clearly decreased the σ_max_ value of PLA to 35.3 MPa and 24.0 MPa, respectively, which is in agreement with previous studies [41,57], whereas the E values remained nearly unaltered, being not significantly different (*p* > 0.05). However, the ε_b_ value also slightly decreased to 6.1% and 4.3% for PLA+OLA2 and PLA+OLA2_mal_, respectively. This result could be due to the fact that both OLAs reduce the cohesion of the macromolecular PLA chains. In this regard, Burgos et al. [57] reported that the E values of PLA decreased with the content of OLAs from 2500 MPa, for neat PLA, to 250 MPa for PLA containing 25 wt.% OLA content. Intermediate values were obtained when OLA2 and OLA2_mal_ were added to the green composite. In particular, the OLA2-containing piece showed the lowest resistant properties, as the E value significantly decreased to 2042 MPa, while σ_max_ slightly, but significantly (*p* < 0.05), increased to 18.9 MPa in relation to the green composite sample. However, it also showed ductile properties, with an ε_b_ value of 33.4%. For the green composite piece with OLA2_mal_, one can observe that it presented better mechanical resistant properties, showing values of E and σ_max_ of 2291 MPa and 20.7 MPa, respectively, but ε_b_ still resulted in a value of 20.7%. This difference observed in the mechanical response of the green composite pieces with the two OLAs tested here can be ascribed to the MAH content of OLA2_mal_, which makes it more reactive and can lead to better chain-to-chain PLA interactions [58]. Moreover, the presence of the multiple MAH groups can provide certain grafting for the cellulose materials onto the backbone of PLA and, thus, acting as an interfacial compatibility agent with the SCG particles [32]. These results were evidenced by the σ_max_ increases of approximately 36% and 60% observed for the PLA+OLA2+SCG and PLA+OLA2_mal_+SCG pieces, respectively, in comparison to the PLA+SCG piece. All in all, the combination of the three materials, namely SCGs, OLA2, and OLA2mal, yielded PLA pieces with varying mechanical performances in terms of rigidity and ductility. Therefore, the simultaneous addition of OLA2 and, more notably, OLA2_mal_ increased the mechanical strength of the green composite PLA+SCG and also provided intermediate ductile properties.

Regarding Shore D hardness, it can be observed that most of the PLA and its green composite pieces developed in the present study showed values in the 80–82 range as being, in most cases, slightly lower than neat PLA. This fact agrees with the results obtained by Lascano et al. [41] and it supports the fact that SCGs, despite being hard lignocellulosic fillers [37], yield an overall softening effect on the PLA material due to their high lipid content. The only significant (*p* < 0.05) change was observed for the green composite containing PLA+OLA2, for which the hardness decreased to 76.8, due to the plasticizing effect exerted by this type of OLA that seems to ascribe more mobility to the PLA chains. In relation to the impact strength, the neat PLA piece showed a value of 27.7 kJ/m^2^, which is a relatively low value, and characteristic of a brittle material [41]. The addition of SCGs at 20 wt.% significantly reduced this value further (*p* < 0.05) to 18.6 kJ/m^2^, which could be attributed to the presence of SCG aggregates that embrittled the PLA matrix by creating local tensions [59]. In this regard, da Silva et al. [60] also reported a toughness decrease in PLA of 6% for contents of SCGs above 15 wt.%. The impact strength of the PLA pieces and the green composite pieces successfully increased after the addition of both OLAs. In the case of the neat PLA, without SCGs, OLA2 increased its impact strength by approximately 6%, while OLA2_mal_ increased it by 12.6%. As reported earlier [41], this slight, but significant (*p* < 0.05), energy absorption enhancement during impact can be ascribed to the high solubility of OLAs in the PLA matrix that enables the inhibition of microcrack formation and growth. However, it is also worth noting that the combination of SCGs with OLA2 and OLA2_mal_ yielded a slight improvement to the overall impact strength of the injection-molded green composite pieces, from which it can be inferred that, for these formulations, the OLAs tested in the present study mainly act as compatibilizers, rather than impact modifiers.

### 3.4. Morphology of the PLA/SCG Composites

**Figure 5:**
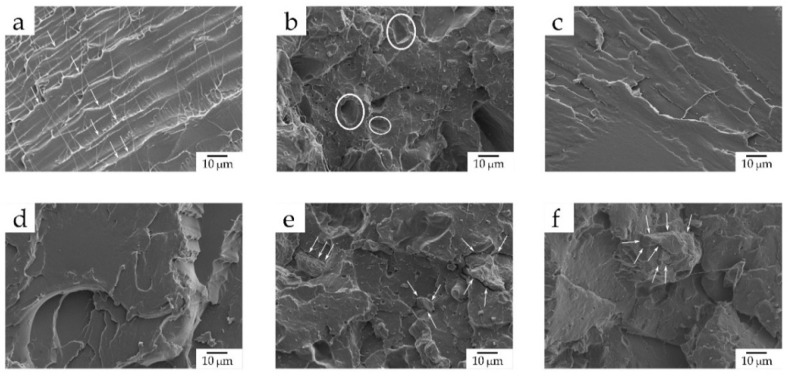
Field emission scanning electron microscopy (FESEM) images, taken at 1000× and with scale markers of 10 µm, of the fracture surfaces of the injection-molded pieces of polylactide (PLA)/spent coffee grounds (SCGs) containing the oligomers of lactic acid (OLAs): (**a**) PLA; (**b**) PLA+SCG; (**c**) PLA + OLA2; (**d**) PLA + OLA2_mal_; (**e**) PLA + OLA2 + SCG; and (**f**) PLA + OLA2_mal_ + SCG.

In order to better ascertain the effect of the SCG particles and the two tested OLAs on PLA, the morphology of the fracture surfaces of the injection-molded pieces obtained after the impact tests were observed by FESEM. The resultant micrographs are presented in Figure 5. As it can be observed in Figure 5a, the neat PLA piece displayed the characteristic morphology of a polymer with brittle behavior due to the formation of a smooth fracture surface with the presence of microcracks (see the white arrows). A similar morphology was previously reported for PLA by Quiles-Carrillo et al. [61], confirming the aforementioned mechanical properties in relation to its low impact strength and ductility. Conversely, in Figure 5b, the fracture surface of the piece changed to a rougher morphology, as a result of the increase in ductility and the presence of the SCG fillers. However, the existence of some holes (identified in the image by white circles) corroborates the lack of compatibility between the PLA matrix and SCGs. These voids correspond to the lignocellulosic particles that were detached after impact [14]. Although a good dispersion of the SCG particles along the PLA matrix can be inferred due to the manifold presence of well-distributed holes, the suggested poor filler-to-matrix adhesion contributed to a reduced mechanical resistance, as shown above, during the mechanical analysis.

Figure 5c,d show the micrographs of the fracture surface for both the PLA+OLA2 and PLA+OLA2_mal_ pieces, respectively, which yield very similar morphologies. In contrast with the fracture surface of the neat PLA piece, which presented some microcracks, one can observe the formation of several macrocracks along the biopolyester matrix. This feature, which was reported by Lascano et al. [41] when studying the effect of different OLA concentrations on PLA, can be appreciated, especially in the fracture surface of the PLA+OLA2_mal_ piece. Therefore, OLA2 and, more noticeably, OLA2_mal_, led to a rougher surface that corresponds to a fracture with a major energy absorption, which agrees with the mechanical properties reported above in terms of toughness. Interestingly, it is also worth mentioning that phase separation was not noticeable due to the great chemical affinity between PLA and OLAs. Finally, Figure 5e,f show the FESEM micrographs corresponding to the fracture surfaces of the PLA+OLA2+SCG and PLA+OLA2_mal_+SCG pieces, respectively. It can be observed that, in both cases, the interfacial adhesion of the SCG particles with the surrounding PLA matrix was noticeably improved due to the decrease in the void density. Furthermore, the SCG particles seem to be more imbedded into the biopolyester, since the gap between the edge of the particles (see the white arrows) and the PLA matrix was very narrow. Thus, both OLAs can potentially increase the affinity of the SCG particles in PLA, especially OLA2_mal_, due to the presence of the multiple MAH groups that can interact with both the backbone ester (R—COO—R) groups of PLA and —OH groups of SCGs [54]. This visual effect was more intense in the PLA+OLA2_mal_+SCG sample, since the particle-matrix gap was almost imperceptible, which correlates better with the mechanical properties reported above.

### 3.5. Thermal Properties of the PLA/SCG Composites

**Figure 6:**
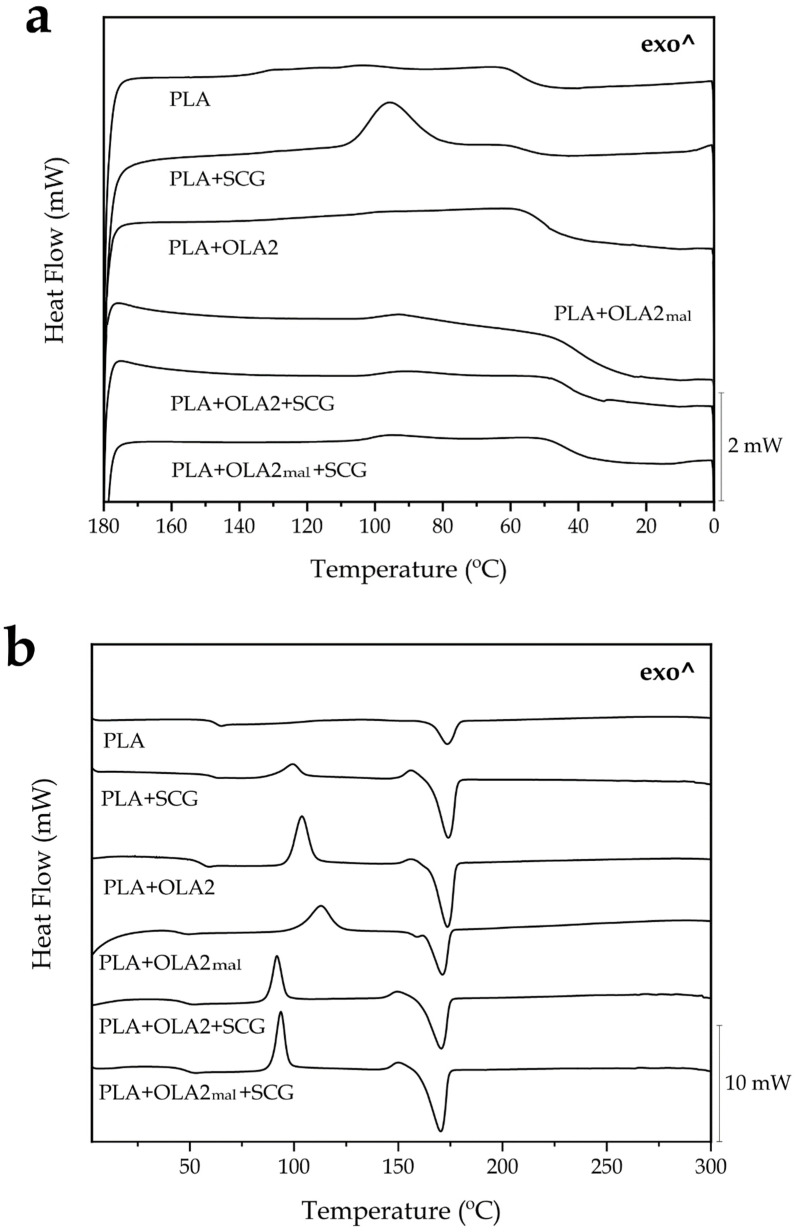
Differential scanning calorimetry (DSC) thermograms of the injection-molded pieces of polylactide (PLA)/spent coffee grounds (SCGs) containing the oligomers of lactic acid (OLAs) during cooling (**a**) and second heating (**b**).

The DSC thermograms corresponding to the cooling and second heating steps were analyzed to study the thermal properties of the green composites. Figure 6 shows the cooling (Figure 6a) and heating curves (Figure 6b), whereas Table 4 gathers the main thermal parameters obtained from these thermograms. One can observe that the neat PLA sample showed a T_g_ of 62.8 °C, which is noticeable from a change in the baseline of the heating thermogram. A very similar result was observed by Lascano et al. [41], who reported a PLA T_g_ value of 63 °C. In this case, however, cold crystallization was not observed during the second heating, due to the fact that complete crystallization was fully achieved during the slow cooling step carried out at 10 °C·min^−1^. During cooling, the PLA molecules crystallized from the melt, showing a low-intense crystallization temperature (T_c_) at nearly 100 °C. The melting of the biopolymer finally occurred at a temperature of 173.3 °C, which is also close to the melting temperature (T_m_) value previously obtained [41]. The addition of SCGs to PLA slightly reduced the value of T_g_, from 62.8 °C to 61.1 °C, supporting the plasticizing effect exerted by the coffee oil on the biopolyester amorphous regions. The green composite sample also crystallized from the melt, showing a T_c_ value of 95.6 °C, whereas it cold crystallized further during the second heating at 99.3 °C, which corresponds to the cold crystallization temperature (T_CC_). The higher intensity of the crystallization peak certainly points to the fact that the presence of SCGs increased the crystallization of the PLA molecules from the melt. Finally, the T_m_ value of the PLA sample was unaffected by the presence of SCGs, showing no significant difference (*p* > 0.05). In relation to the crystallinity, the neat PLA sample showed an X_C_ value of nearly 20%, which is very similar to that obtained by Rojas-Lema et al. [62], whereas the single addition of SCGs increased the crystallinity to approximately 36%. In the case of the maximum crystallization degree (X_C_max_), which does not consider the crystal formed during heating and presents more accurate information about the effect of a given additive on the polymer crystallinity, this increase was higher, nearly 54%. This result indicates that the lignocellulosic fillers acted as heterogenous nuclei during the formation of the crystals, and considerably increased the number of crystals that were formed. This phenomenon was previously described by da Silva et al. [60], who also observed an increase in the PLA crystallinity by means of SCGs obtained as waste from the preparation of instant coffee. The authors reported an increase in the crystallinity of PLA from 9.10% to 12.7% for a 5 wt.% content of SCGs in the green composite.

In regard to the effect of the OLAs, it can be observed that OLA2 and OLA2_mal_ provoked a significant (*p* < 0.05) decrease in T_g_, reducing it to 55.4 °C and 45.33 °C, respectively. This reduction has been attributed to the plasticizing effect exerted by OLAs [42,58]. Similar to the case of SCGs, for the samples containing OLA2 and OLA2_mal_, cold crystallization also occurred, showing T_CC_ values of 103.7 °C and 112.9 °C, respectively. This suggests that the PLA crystallization was promoted, as a result of its high solubility and interaction with the PLA molecules [60]. In terms of melting, the slightly lower T_m_ value attained in the OLA2_mal_-containing sample, which was reduced to 171 °C, confirms its higher interaction with the PLA matrix. However, although the crystallinity degree, that is, X_C_, was not significantly (*p* > 0.05) affected by the addition of both OLAs, one can observe that the maximum crystallinity degree, that is, X_C_max_, increased to approximately 51% and 42%, for OLA2 and OLA2_mal_, respectively. In this regard, other previous studies reported that OLAs can also act as heterogeneous nucleating agents and facilitate the crystal formation in PLA, since they are still present in the form of thin solids with a melting point higher than the T_C_ value of PLA [63]. In regard to the combined additions of SCGs and the two tested OLAs, one can observe that T_g_ suffered a considerable decrease due to the plasticization of PLA by both the coffee oil and the presence of the oligomers. The T_CC_ values also decreased further to 91.9 °C and 93.7 °C for PLA+OLA2+SCG and PLA+OLA2_mal_+SCG, respectively, which can be ascribed to the facilitated mobility of the amorphous phase. However, even though the PLA matrix was highly plasticized, the absence of crystallization from the melt may also suggest an impairment during the crystal formation due to the presence of these additives. In all cases, and similar to the PLA+SCG, PLA+OLA2, and PLA+OLA2_mal_ samples, the green composites containing both additives showed significantly (*p* < 0.05) higher values of X_C_max_, with values of approximately 54% and 61% for PLA+OLA2+SCG and PLA+OLA2_mal_+SCG, respectively.

**Figure 7:**
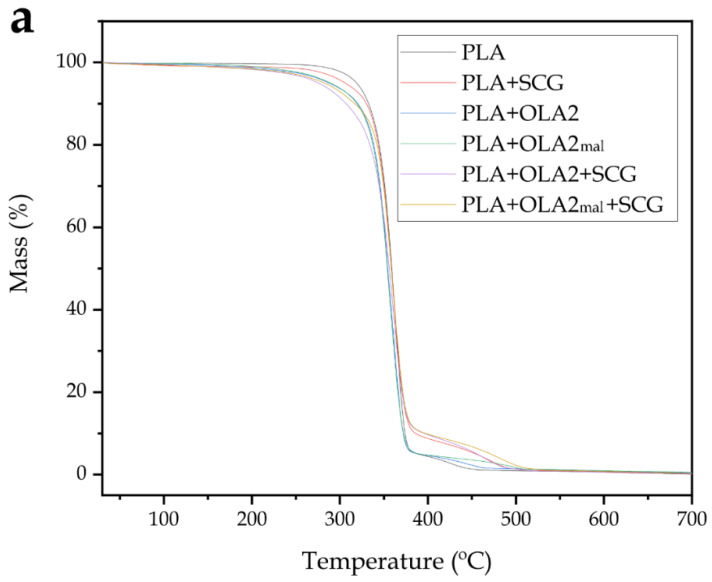

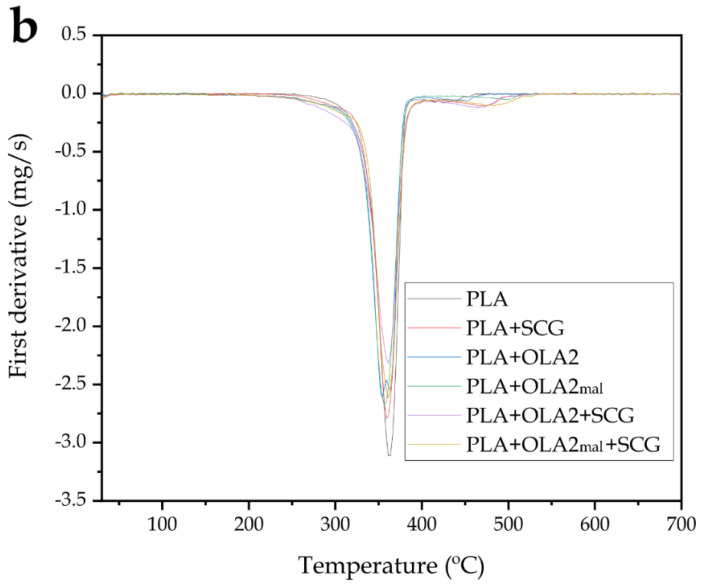
(**a**) Thermogravimetric analysis (TGA) curves and (**b**) first derivative (DTG) of the injection-molded pieces of polylactide (PLA)/spent coffee grounds (SCGs) containing the oligomers of lactic acid (OLAs).

In terms of the thermogravimetric characterization, Figure 7 presents the variations of mass (Figure 7a) and the first derivatives (Figure 7b) with temperature to evaluate the degradation processes of each sample. Moreover, Table 5 gathers and presents the most relevant parameters related to the degradation processes. In this sense, it can be observed that neat PLA exhibited a single-step degradation process, showing a slightly superior thermal stability than its green composites with SCGs and blends with OLAs. In particular, PLA presented the values of T_5%_ and T_deg_ of 319.3 °C and 361.3 °C, showing a mass loss at T_deg_ of 57.2%. A very similar degradation profile was observed for PLA by Rojas-Lema et al. [62]. The addition of SCGs significantly (*p* < 0.05) reduced the thermal stability of PLA and generated a low-intense second degradation step at high temperatures. The first and main weight loss initiated at approximately 305 °C, which means a reduction of approximately 14 °C with respect to the neat PLA. This is related to the degradation of low-M_W_ components present in the lignocellulosic residue, such as hemicellulose [64]. The second step, which appeared at the 370–500 °C range, was less pronounced and it can be ascribed to the thermal degradation of lignin and cellulose present in SCGs [65]. The maximum degradation peak, represented by T_deg_, also decreased by approximately 2 °C versus the neat PLA, although this difference was not significant (*p* > 0.05). A similar reduction in the thermal stability was observed by Mendes et al. [46] when studying the thermal properties of HDPE/SCG composites. The incorporation of OLA2 and OLA2_mal_ into PLA also decreased the onset degradation, that is, T_5%_, to 287.7 °C and 289 °C, respectively. In the case of T_deg_, a reduction to the values of 354.3 °C and 356.7 °C was, respectively, observed, meaning a significant (*p* < 0.05) loss of thermal stability. In this context, several studies reported the inherently lower thermal stability of OLAs in comparison to PLA [41,62]. Furthermore, OLA2_mal_ yielded a slightly higher thermal stability than OLA2. This fact can be ascribed to the presence of MAH groups in this OLA grade that, similar to other properties, resulted in a higher interaction with the PLA matrix by forming a more stable macromolecule, in which the chain–scission process at a high temperature was delayed [58]. The combined systems of PLA+OLA2+SCG and PLA+OLA2_mal_+SCG also showed the characteristic two-step degradation process of the green composite due to the presence of the lignocellulosic filler. Thermal degradation occurred more rapidly at the start of the process, showing T_5%_ values of 275 °C and 282 °C, respectively. However, the T_deg_ values were not significantly different (*p* > 0.05) than those of the neat PLA, that is, 361.3 °C, being slightly higher than those of the green composite without OLAs and the OLA-containing PLA samples. Finally, the residual mass, in all cases, was below 1 wt.%. This suggests a good synergetic effect between SCGs and OLAs, which was previously observed during the morphological analysis. In general terms, one can thus consider that the combined addition of SCGs derived from the liquor industry and OLAs can lead to materials with a relatively similar thermal stability than PLA, which are adequate for applications that deal with temperatures below 275 °C.

### 3.6. Thermomechanical Properties of the PLA/SCG Composites

**Figure 8:**
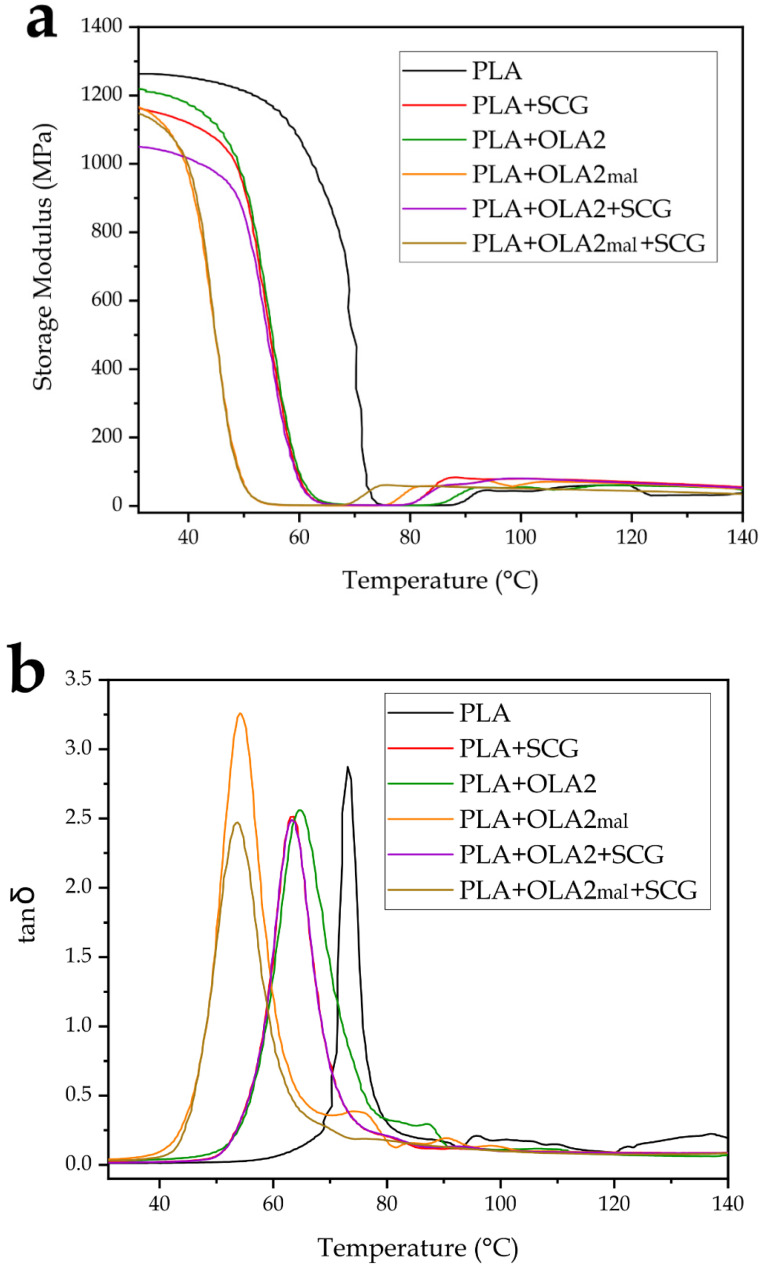
Plot evolution of (**a**) the storage modulus and (**b**) the dynamic damping factor (tan δ) of the injection-molded pieces of polylactide (PLA)/spent coffee grounds (SCGs) containing the oligomers of lactic acid (OLAs).

DMTA determines the mechanical properties as a function of temperature in dynamic conditions through the application of sinusoidal stress. Figure 8 shows the DMTA curves for all the PLA/SCG composites, while Table 6 gathers the most relevant thermomechanical parameters. In Figure 8a, one can observe the evolution of the storage modulus as a function of temperature. Regarding the neat PLA piece, the storage modulus suffers a pronounced drop in the temperature range between 50 °C and 70 °C, which is indicative of the α-relaxation process of the PLA chains when surpassing the glass transition region [41]. This can be observed in the difference between the storage modulus of 1263 MPa, at 35 °C, and the storage modulus value of 1.5 MPa, at 80 °C. Another important feature is the storage modulus change in the 90–100 °C range, which corresponds to the cold crystallization process [37]. This provoked a rearrangement of the PLA chains into a more ordered structure with higher thermomechanical resistance. The addition of SCGs to PLA significantly (*p* < 0.05) decreased the storage modulus of the biopolyester to 1150 MPa at 35 °C, which can be ascribed to the plasticization process by the remaining coffee oil in the filler. However, it yielded the cold crystallization process to occur at lower temperatures than in the case of the neat PLA (80–90 °C range). This confirms that the SCG particles also acted as nucleating agents during the crystallization process of PLA, as previously described during the DSC analysis, resulting in a storage modulus of 80 MPa at 100 °C. Similar behavior was observed by Quiles-Carrillo et al. [37] when PLA was reinforced with almond shell flour (ASF), a lignocellulosic filler. With the incorporation of OLA2 into the PLA matrix, the storage modulus presented a value of 1210 MPa at 35 °C, which is slightly, but still significantly (*p* < 0.05), lower than the value for PLA. Furthermore, in comparison with neat PLA, the storage modulus values of the OLA-containing PLA pieces were lower along the whole temperature range up to the glass transition region of the biopolymer, where they were exceeded. This result confirms that OLA2 reduces the stiffness of PLA due to its role as a plasticizing agent, as it was previously mentioned during the mechanical analysis. Similar behavior was observed in the OLA2_mal_ sample, but with a more emphasized effect, reducing the storage modulus to 1130 MPa at 35 °C. It should also be noted that both OLAs reduced the temperature at which the cold crystallization phenomenon occurs. This is representative of the nucleating effect provided by the short-length OLA molecules, which were able to facilitate the packing of the PLA macromolecular structure [63]. The combination of OLA2 and OLA2_mal_ with SCGs also led to a decrease in the storage modulus in all the temperature ranges, which can be mainly ascribed to the plasticizing effect of both the coffee oil and oligomers. The T_CC_ value was also reduced as a result of the combined nucleating effect of SCGs and OLA2 or OLA2_mal_, which was previously observed during the DSC analysis. Moreover, during the thermomechanical analysis, it was observed that OLA2_mal_ exerted a higher nucleating effect, as it decreased the crystallization process to the 70–80 °C temperature range.

Figure 8b presents the evolution of the dynamic damping factor (tan δ) with the temperature. This allows us to identify the α-relaxation of PLA, which is related to its T_g_, observed as the maximum peak. The neat PLA shows a tan δ peak at 73.1 °C, which agrees with the previous values reported by the other authors [37,41]. The presence of SCGs in the PLA matrix decreased the tan δ peak to 63.1 °C due to the previously described plasticization process by the coffee oil, providing it with improved chain mobility and free volume. Moreover, it can be observed that OLA2 and OLA2_mal_ also reduced the tan δ peaks to 64.9 and 54.4 °C, respectively, which successfully correlates with the T_g_ values attained in the DSC results shown above. Thus, the combined addition of OLA2 or OLA2_mal_ with SCGs followed the same tendency as the single ones for OLA2 or OLA2_mal_. In particular, the PLA+OLA2_mal_+SCG sample presented a very similar tan δ peak value than the OLA2_mal_ sample, that is, 53.5 °C, while the PLA+OLA2+SCG and PLA+OLA2 pieces presented values that exceeded 60 °C, specifically 63.2 °C in the case of the green composite. Interestingly, all of the green composite samples presented a maximum damping factor that was approximately 15% lower than in the unfilled PLA samples. This reduction is ascribed to the replacement in the injection-molded piece of the biopolymer with an amorphous region with a hard lignocellulosic filler, by which the material presents a reduced energy dissipation and toughness [36].

### 3.7. Water-Resistance Properties of the PLA/SCG Composites

**Figure 9:**
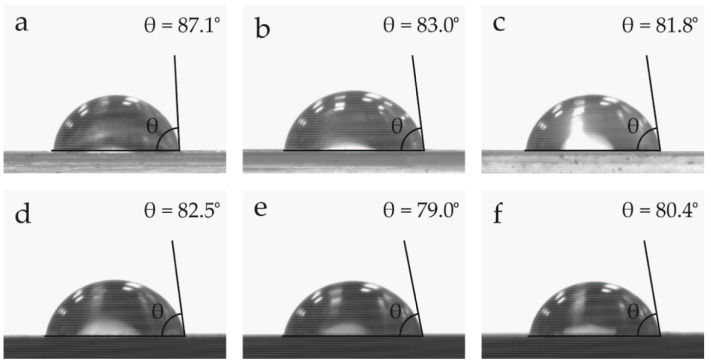
Water contact angle of the injection-molded pieces of polylactide (PLA)/spent coffee grounds (SCGs) containing the oligomers of lactic acid (OLAs): (**a**) PLA; (**b**) PLA + OLA2; (**c**) PLA + OLA2_mal_; (**d**) PLA + SCG; (**e**) PLA + OLA2 + SCG; and (**f**) PLA + OLA2_mal_ + SCG.

Water contact angle measurements were conducted in order to obtain information about the hydrophilic behavior of the sample pieces and to evaluate the effect of the additives on the material surface. Figure 9 presents the water drops and their respective contact angles with the surface of the PLA pieces. It can be observed that all of the samples showed hydrophilicity since their contact angle was less than 90°. In particular, the neat PLA piece showed a contact angle of 87.1°, which is almost in the 90° limit and characteristic of the hydrophobic behavior of the biopolyester [66,67]. The addition of OLA2 and OLA2_mal_ reduced the water contact angle to 83° and 81.8°, respectively, and thus made PLA more hydrophilic. This is due to the more hydrophilic nature of OLAs in comparison to PLA, as reported by Darie-Niţă et al. [66] when they studied different plasticizers for processing PLA films. In particular, the authors showed a reduction of about 12.5% in the contact angle in relation to the neat PLA for a 7 wt.% content of oligomers. In regard to the incorporation of the SCG particles, one can observe that the hydrophilicity of PLA also increased, reducing the value of the contact angle to 82.5°. This reduction can be related to the higher hydrophilicity of SCGs due to the high-content –OH groups in the lignocellulosic particles. This effect was previously observed by Laaziz et al. [67] when filling PLA with lignocellulosic almond particles. The combined additions that resulted in the PLA/OLA2/SCG and PLA/OLA2_mal_/SCG pieces further decreased the contact angle to 79° and 80.4°, respectively. This is indicative of a synergistic effect between SCGs and OLAs, which both increase the polarity of PLA and its free volume. Moreover, it should be noted that the differences observed for the OLA2- and OLA2_mal_-containing pieces can be related to their composition. In particular, it can be considered that OLA2_mal_ achieved a lower contact angle value due to the presence of MAH, which increases the polarity of PLA to a higher extent than OLA2.

**Figure 10:**
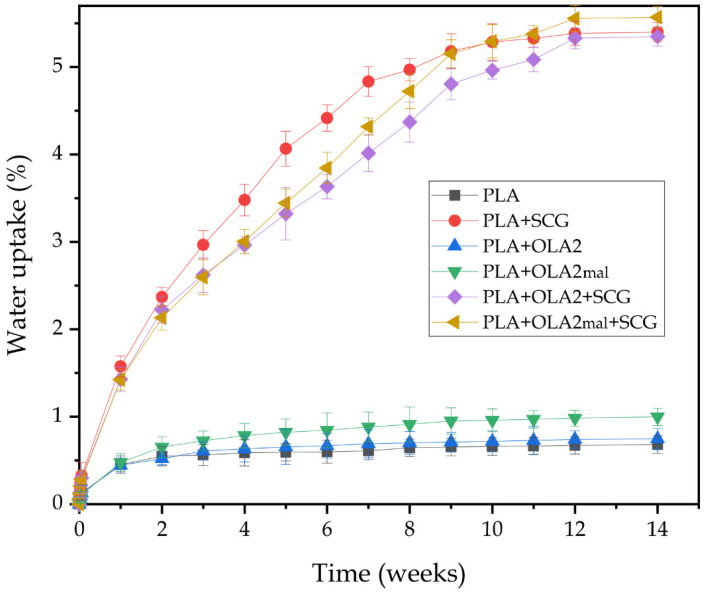
Water uptake of the injection-molded pieces of polylactide (PLA)/spent coffee grounds (SCGs) containing the oligomers of lactic acid (OLAs).

In addition to the contact angle measurements, the water uptake of the injection-molded pieces was evaluated through the water absorption test. Figure 10 presents the evolution of the water uptake of each sample after 14 weeks of immersion in distilled water. As expected, the neat PLA piece exhibited the lowest water absorption value during all of the immersion times, with a saturation value of approximately 0.67 wt.% at 14 weeks of immersion due to the hydrophobic nature of PLA. In this regard, Balart et al. [68] observed a very similar saturation content of water for PLA. The addition of SCGs to the PLA matrix significantly increased the water absorption capacity of the piece to a saturation value of 5.38 wt.%, which is more than 5 times the water absorption value of the neat PLA. This high increase is related to the hydrophilic nature of SCGs, as shown above during the water contact angle. In this sense, cellulose, hemicellulose, and lignin, all present in SCGs, contain multiple –OH groups that can readily interact with water molecules, forming hydrogen bonds and allowing water to enter the PLA’s structure [68]. Similarly, Wu [32] reported high water absorption values for the PLA/SCG composites, reaching about a 12.5 wt.% water absorption value for a 20 wt.% filler content after 60 days of immersion in water. On the other hand, it is worth mentioning that the presence of OLA2 and OLA2_mal_ in the PLA matrix slightly increased the water uptake to values of 0.74 wt.% and 0.98 wt.%, respectively. This small increase may be related to the higher free volume attained in the PLA samples due to the plasticization caused by the oligomers, being slightly higher for OLA2_mal_ due to its higher interaction with the biopolymer matrix as a result of the presence of the MAH groups, which are highly hydrophilic. Similar behavior was observed by Wu [32], who treated PLA with MAH and observed a water absorption value that was superior by approximately 4% to that of the neat PLA. Finally, the combined additions in the PLA+OLA2+SCG and PLA+OLA2_mal_+SCG pieces yielded saturation values of 5.33 and 5.55 wt.%, respectively, which are very similar to the value obtained for the PLA+SCG piece. However, the water uptake rate was lower, particularly from week 2, which corroborates the above-reported morphological results in which it was indicated that the OLAs were mainly located at the filler-to-matrix interface and, then, could potentially reduce the water absorption on the lignocellulosic particle surfaces.

### 3.8. Disintegration in Controlled Compost Soil of the PLA/SCG Composites

**Figure 11:**
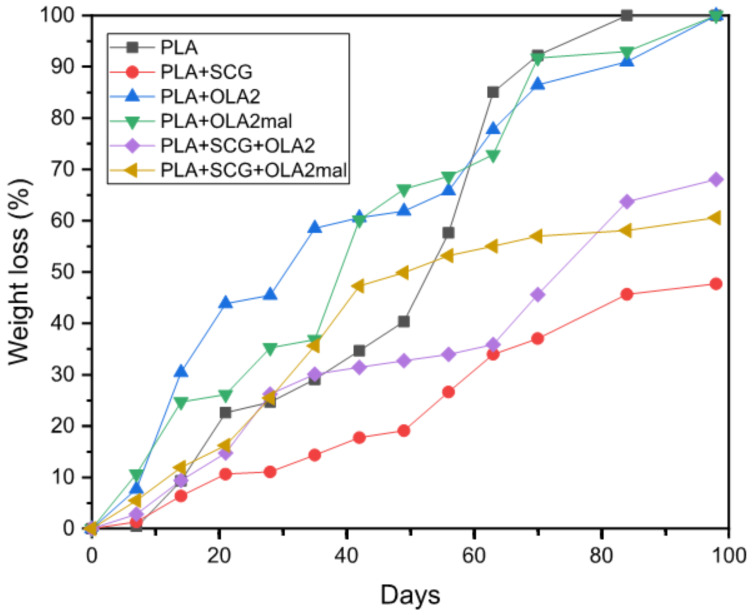
Evolution plot of the percentage of weight loss as a function of the elapsed time during disintegration in the controlled compost soil of the injection-molded pieces of polylactide (PLA)/spent coffee grounds (SCGs) containing the oligomers of lactic acid (OLAs).

Finally, the effect of both SCGs and OLAs on the biodegradation rate of PLA was ascertained. Figure 11 shows the percentage of weight loss as a function of the elapsed time during disintegration in the controlled compost soil of the injection-molded pieces. It can be observed that only the PLA, PLA+OLA2, and PLA+OLA2_mal_ samples fully disintegrated during the test. This fact is related to the large thickness of the PLA pieces, that is, 4 mm. Nonetheless, all of the pieces achieved a high weight loss after 98 days in compost soil. The neat PLA presented a weight loss of 100% after 84 days due to a hydrolytic degradation process [68]. A similar disintegration profile was observed by Quiles-Carrillo et al. [69] when studying the compostability of PLA, achieving a mass decomposition of 100 wt.% after 56 days. These differences can be explained in terms of the compost composition and conditions. One can observe that the addition of 20 wt.% of SCGs highly decreased the biodegradation rate in comparison to the neat PLA, showing a weight loss of approximately 48% after 98 days. This delay in the disintegration rate can be due to the higher crystallinity that the PLA/SCG samples possess in relation to the neat PLA, as shown above during the DSC analysis. Since hydrolytic degradation occurs more rapidly in the amorphous regions thus, the higher the crystallinity, the lower the disintegration rate. In this regard, Balart et al. [68] also observed this effect for PLA/hazelnut shell flour (HSF) composites, where the lignocellulosic filler delayed the disintegration of PLA. The inclusion of OLA2 and OLA2_mal_ into the PLA matrix increased the disintegration rate of the pieces during the first weeks, which could be ascribed to the fact that both oligomers plasticized the amorphous region of PLA and increased the free volume, favoring the hydrolysis of the biopolyester chains. Thus, after 49 days, PLA/OLA2 and PLA/OLA2_mal_ presented a mass loss of approximately 60% and 65%, respectively, which was more rapid in comparison to the neat PLA that biodegraded by 55%. Finally, the combined additions of PLA with SCGs and OLA2 and OLA2_mal_ showed disintegration rates lower than that of the neat PLA, presenting mass losses of nearly 65% and 60% at 98 days, respectively, and not fully degrading. This can be ascribed to the higher crystallinity of the green composite samples. However, their disintegration was superior to that observed for the PLA+SCG piece, because of the plasticization process.

**Figure 12:**
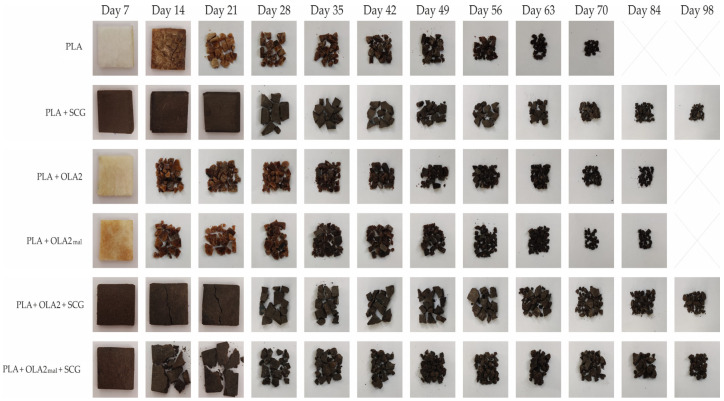
Visual aspect at selected disintegration times of the injection-molded pieces of polylactide (PLA)/spent coffee grounds (SCGs) containing the oligomers of lactic acid (OLAs).

Finally, Figure 12 shows the visual aspect of all the PLA pieces studied during the disintegration test, providing more information about their compostability profile. From the visual appearance of these samples, it can be confirmed that the SCG-containing pieces were not fully disintegrated after being buried for 98 days in compost soil. In regard to the neat PLA, it lost its transparent appearance only after 7 days of incubation in compost soil due to the hydrolysis process of the biopolyester [70]. After 14 days, the PLA pieces already started to develop a brown color, which evidences the beginning of disintegration. It then started to fragment and present microcracks, until its complete biodisintegration after 98 days. The PLA+SCG pieces did not present apparent disintegration signs until day 28, when it cracked and then started to fragment. It can also be observed that their disintegration rate was slower than that of PLA. Regarding the PLA+OLA2 and PLA+OLA2_mal_ pieces, both started to show a dark brown color after 14 days, being quite fragmented at this time, which confirms the more rapid disintegration rate in comparison to the neat PLA. This confirms the faster disintegration profile observed in Figure 11, in which the two OLA-containing samples displayed a higher disintegration rate than neat PLA during the first 56 days, although the latter piece fully disintegrated earlier on. Concerning the simultaneous additions to produce the so-called PLA+SCG+OLA2 and PLA+SCG+OLA2_mal_ pieces, these yielded similar disintegrated samples. However, the PLA+SCG+OLA2_mal_ piece started to fragment earlier (day 14), in agreement with the disintegration profile shown above. These results suggest that, even though PLA is already a biodegradable polymer under compost soil conditions, the addition of OLAs can promote its disintegration. On the other hand, the incorporation of SCGs significantly delayed the biodisintegration of PLA, a fact that can be closely linked with the increase in the crystallinity that this food waste derived lignocellulosic filler provides.

## 4. Conclusions

The successful transition in the packaging industry of linear economies to circular and more sustainable bioeconomies certainly requires the development of high-performance materials derived from biomass and industrial wastes. The results presented in this work demonstrate, for the first time, the high potential of manufacturing PLA-based green composites through the valorization of wastes derived from the liquor coffee industry, referred to in the present study as SCGs. The use of these lignocellulosic fillers yielded PLA materials with an enhanced ductility and potentially lower cost due to the use of a residue with a low economical value. In terms of the mechanical performance, the addition of the liquor waste derived SCGs increased the ε_b_ value of the injection-molded PLA pieces from 10.4% to 39.6%, which represents an improvement of approximately 280%. This enhancement was related to the higher lipid content and remaining solvent traces present in the coffee wastes derived from the liquor preparation. In this industry, coffee grains are naturally roasted and then macerated with ethanol to obtain alcoholic beverages, so that they are not subjected to extraction processes with high temperatures. Furthermore, the simultaneous addition of OLAs, particularly the oligomer functionalized with multiple MAH groups, contributed to improve the impact strength and, more notably, the tensile strength of the green composite pieces. In particular, the OLA2_mal_ sample yielded a σ_max_ value of 20.7 MPa, respectively, corresponding to improvements of nearly 60% in relation to the PLA/SCG sample. The results attained in the present study were ascribed to the nucleation effect and interaction of the SCG fillers with the PLA matrix due to the presence of the multiple MAH groups in the oligomer, narrowing the filler-to-matrix gaps. In all cases, a relatively high thermal stability was attained, being adequate for applications that deal with temperatures below 275 °C, whereas the visual appearance of the pieces showed a typical dark-brown color that can resemble natural materials, such as wood. Finally, the resultant green composites showed slightly lower disintegration rates than PLA, but still biodegraded in compost conditions.

Therefore, it can be concluded that the use of coffee waste derived from the liquor industry is very promising for developing cost-effective green composites and it opens a new route towards the development of ductile PLA-based materials. These novel green composites are characterized by showing moderate tensile strength and ductility, thermal stability, being renewable and inexpensive due to being based on biomass derived from food wastes, and also biodegradable in industrial composting facilities. All of these features can open the door to their use in disposable food-serving utensils and tableware, such as plates, trays, spoons, forks, knives, cups, or straws. Moreover, a bright vision for the future might be anticipated for their applications in sustainable food packaging and related uses, for instance, single-use coffee cups and capsules that can be organically recyclable to reduce environmental pollution. Future works will focus on further optimizing the amount of SCGs, the development of other compatibilizers that lead to a higher performance, and also on the evaluation of the active properties of the resultant composites, such as their antioxidant capacity for food preservation. Moreover, the organoleptic properties and stability of the green composites over time will also be explored.

## Figures and Tables

**Table 1:** Summary of the compositions according to the weight content (wt.%) of polylactide (PLA), spent coffee grounds (SCGs), and oligomers of lactic acid (OLAs).

Piece	PLA (wt.%)	SCG (wt.%)	OLA2 (wt.%)	OLA2_mal_ (wt.%)
PLA	100	0	0	0
PLA + SCG	80	20	0	0
PLA + OLA2	90	0	10	0
PLA + OLA2_mal_	90	0	0	10
PLA + OLA2 + SCG	70	20	10	0
PLA + OLA2_mal_ + SCG	70	20	0	10

**Table 2:** Luminance and color coordinates (L*a*b*) of the injection-molded pieces of polylactide (PLA)/spent coffee grounds (SCGs) containing the oligomers of lactic acid (OLAs).

Piece	L*	a*	b*
PLA	48.0 ± 0.5 ^a^	−0.23 ± 0.02 ^a^	1.89 ± 0.08 ^a^
PLA + SCG	26.6 ± 0.1 ^b^	1.10 ± 0.13 ^b^	5.42 ± 0.19 ^b^
PLA + OLA2	45.6 ± 0.1 ^c^	0.13 ± 0.02 ^c^	1.25 ± 0.08 ^c^
PLA + OLA2_mal_	47.4 ± 0.2 ^c^	−0.14 ± 0.02 ^d^	1.68 ± 0.09 ^d^
PLA + OLA2 + SCG	26.3 ± 0.3 ^b^	0.74 ± 0.02 ^e^	4.91 ± 0.06 ^e^
PLA + OLA2_mal_ + SCG	25.8 ± 0.2 ^b^	0.75 ± 0.04 ^e^	5.56 ± 0.11 ^f^

^a–f^ Different letters in the same column indicate a significant difference among the samples (*p* < 0.05).

**Table 3:** Mechanical properties of the injection-molded pieces of polylactide (PLA)/spent coffee grounds (SCGs) containing the oligomers of lactic acid (OLAs) in terms of tensile modulus (E), maximum tensile strength (σ_max_), elongation at break (ε_b_), Shore D hardness, and impact strength.

Piece	E (MPa)	σ_max_ (MPa)	ε_b_ (%)	Shore D Hardness	Impact Strength (kJ·m^−2^)
PLA	2913 ± 84 ^a^	52.4 ± 3.1 ^a^	10.4 ± 0.8 ^a^	82.0 ± 0.5 ^a^	27.7 ± 2.3 ^a^
PLA + SCG	2367 ± 61 ^b^	13.9 ± 0.7 ^b^	39.6 ± 2.0 ^b^	80.6 ± 0.9 ^a^	18.6 ± 1.9 ^b^
PLA + OLA2	2835 ± 49 ^a^	35.3 ± 3.9 ^c^	6.1 ± 0.4 ^c^	76.8 ± 1.3 ^b^	29.3 ± 3.6 ^c^
PLA + OLA2_mal_	3002 ± 172 ^a^	24.0 ± 3.7 ^d^	4.3 ± 0.6 ^d^	82.8 ± 0.8 ^a^	31.2 ± 2.1 ^d^
PLA + OLA2 + SCG	2042 ± 43 ^c^	18.9 ± 1.3 ^e^	33.4 ± 3.0 ^e^	81.4 ± 1.1 ^a^	20.6 ± 2.2 ^e^
PLA + OLA2_mal_ + SCG	2291 ± 96 ^d^	22.2 ± 0.9 ^f^	20.7 ± 2.3 ^f^	81.8 ± 1.1 ^a^	18.9 ± 2.4 ^b^

^a–f^ Different letters in the same column indicate a significant difference among the samples (*p* < 0.05).

**Table 4:** Thermal properties of the injection-molded pieces of polylactide (PLA)/spent coffee grounds (SCGs) containing the oligomers of lactic acid (OLAs) in terms of glass transition temperature (T_g_), crystallization temperature (T_C_), cold crystallization temperature (T_CC_), melting temperature (T_m_), crystallinity (X_C_), and maximum crystallinity (X_C_max_).

Piece	T_g_ (°C)	T_C_ (°C)	T_CC_ (°C)	T_m_ (°C)	X_C_ (%)	X_C_max_ (%)
PLA	62.8 ± 0.2 ^a^	103.7± 0.1 ^a^	-	173.3 ± 1.9 ^a^	19.96 ± 0.3 ^a^	19.96 ± 0.3 ^a^
PLA + SCG	61.1 ± 0.3 ^a^	95.6 ± 1.0 ^b^	99.3 ± 0.9 ^a^	173.6 ± 2.0 ^a^	36.42 ± 0.7 ^b^	53.90 ± 0.6 ^b^
PLA + OLA2	55.4 ± 0.2 ^b^	96.7 ± 0.5 ^b^	103.7 ± 1.2 ^a^	173.3 ± 1.9 ^a^	7.59 ± 0.5 ^c^	50.87 ± 0.5 ^c^
PLA + OLA2_mal_	45.3 ± 0.4 ^c^	94.9 ± 0.2 ^b^	112.9 ± 1.5 ^b^	171.0 ± 2.4 ^a^	3.91 ± 0.4 ^d^	41.98 ± 0.3 ^d^
PLA + OLA2 + SCG	47.3 ± 0.3 ^c^	90.7 ± 0.3 ^c^	91.9 ± 1.1 ^c^	170.2 ± 2.5 ^a^	21.04 ± 0.3 ^e^	54.28 ± 0.1 ^e^
PLA + OLA2_mal_ + SCG	48.4 ± 0.2 ^c^	100.7 ± 0.4 ^d^	93.7 ± 1.0 ^c^	170.1 ± 2.2 ^a^	23.02 ± 0.2 ^f^	61.44 ± 0.2 ^f^

^a–f^ The different letters in the same column indicate a significant difference among the samples (*p* < 0.05).

**Table 5:** Main thermal degradation parameters of the injection-molded pieces of polylactide (PLA)/spent coffee grounds (SCGs) containing the oligomers of lactic acid (OLAs) in terms of the onset degradation temperature measured at a mass loss of 5 wt.% (T_5%_), degradation temperature (T_deg_), mass loss at T_deg_, and residual mass at 700 °C.

Piece	T_5%_ (°C)	T_deg_ (°C)	Mass Loss at T_deg_ (%)	Residual Weight (%)
PLA	319.3 ± 0.8 ^a^	361.3 ± 2.4 ^a^	57.2 ± 0.9 ^a^	0.1 ± 0.1 ^a^
PLA + SCG	305.3 ± 1.1 ^a^	359.0 ± 1.3 ^a^	51.4 ± 1.1 ^b^	0.3 ± 0.1 ^a^
PLA + OLA2	287.7 ± 1.4 ^b^	354.3 ± 1.8 ^b^	49.9 ± 1.0 ^b^	0.4 ± 0.2 ^a^
PLA + OLA2_mal_	289.0 ± 0.5 ^b^	356.7 ± 2.2 ^b^	57.3 ± 0.8 ^c^	0.6 ± 0.2 ^b^
PLA + OLA2 + SCG	275.0 ± 1.2 ^c^	361.3 ± 1.0 ^a^	61.7 ± 1.0 ^d^	0.2 ± 0.1 ^a^
PLA + OLA2_mal_ + SCG	282.0 ± 0.7 ^c^	361.3 ± 1.5 ^a^	57.3 ± 0.7 ^e^	0.5 ± 0.1 ^b^

^a–e^ The different letters in the same column indicate a significant difference among the samples (*p* < 0.05).

**Table 6:** Thermomechanical properties of the injection-molded pieces of polylactide (PLA)/spent coffee grounds (SCGs) containing the oligomers of lactic acid (OLAs) in terms of the storage modulus at 35 °C, 80 °C, and 100 °C, and the dynamic damping factor (tan δ) peak.

Piece	Storage Modulus at 35 °C (MPa)	Storage Modulus at 80 °C (Mpa)	Storage Modulus at 100 °C (Mpa)	Tan δ Peak (°C)
PLA	1263 ± 30 ^a^	1.5 ± 0.1 ^a^	43 ± 5 ^a^	73.1 ± 1.2 ^a^
PLA + SCG	1150 ± 25 ^b^	4.0 ± 0.2 ^b^	80 ± 10 ^b^	63.1 ± 1.4 ^b^
PLA + OLA2	1210 ± 39 ^c^	1.3 ± 0.2 ^a^	53 ± 7 ^c^	64.9 ± 2.5 ^b^
PLA + OLA2_mal_	1130 ± 31 ^b^	58.0 ± 2.0 ^c^	62 ± 4 ^d^	54.4 ± 3.1 ^c^
PLA + OLA2 + SCG	1037 ± 37 ^d^	3.8 ± 0.3 ^b^	80 ± 8 ^b^	63.2 ± 1.7 ^b^
PLA + OLA2_mal_ + SCG	1115 ± 40 ^b^	57.1 ± 2.5 ^c^	51 ± 3 ^c^	53.5 ± 1.6 ^c^

^a–d^ The different letters in the same column indicate a significant difference among the samples (*p* < 0.05).

## Data Availability

Data is contained within the article and also available on request.
